# Hypopharyngeal spindle cell lipoma: A case report and review of literature

**DOI:** 10.1097/MD.0000000000025782

**Published:** 2021-05-07

**Authors:** Zheng Liang, Yiqing Zang, Zhibin Jing, Yujie Zhang, Hui Cao, Huifang Zhou

**Affiliations:** a Department of Otorhinolaryngology; b Department of Pathology, Tianjin Medical University General Hospital, Tianjin, PR China.

**Keywords:** head and neck tumor, rare diseases, spindle cell lipoma

## Abstract

**Rationale:**

Spindle cell lipoma is a rare, uncommon type of benign lipomatous tumor, a distinct group of lipomas composed of mature adipocytes, uniform spindle cells, and multinucleated giant cells associated with ropey collagen. Immunohistochemically, spindle cell lipoma is characterized by the diffuse expression of CD34.

**Patient concerns:**

We present a rare case of a 56-year-old man who complained of vomiting out of a smooth and giant mass in the oral cavity provoked by an intra-abdominal pressure increase. Oral examination revealed an elongated mass protruding from the mouth. Computed tomography of the patient showed a mass from left pyriform to oral cavity, with 2.38 × 2.78 × 16.86 cm in size. The flexible fiberscope showed that the pedicle of the elongated mass originated from the posterior wall of the hypopharynx, corresponding to the left pyriform fossa.

**Diagnosis:**

Histopathologically, the tumor was mainly composed of hyperplastic adipocytes, admixed with small blood vessels, and scattered inside adipose tissue spindle cells. The immunohistochemical profile revealed positivity of spindle cells for CD34, negativity for S100, and low proliferation with Ki67, which confirmed the diagnosis of spindle cell lipoma and revealed its benign behavior.

**Interventions:**

The patient underwent hypopharyngeal mass resection using transoral suspension laryngoscopy.

**Outcomes:**

No recurrence was found after 5 months of follow-up.

**Lessons:**

Spindle cell lipoma is difficult to diagnose early because of slow growth and subtle symptomatology. This entity should be differentiated from several benign or malignant subtypes of lipomas, including liposarcomas. In this case, the spindle cell lipoma is large and originates from the hypopharynx, which is a rare entity and presents with atypical symptoms. This case gave rise to further studies on the clinical and pathologic characteristics of this tumor in the future.

## 1 Introduction

Spindle cell lipoma was first defined by Enzinger et al in 1975. It is a special type of lipoma and is easily mistaken for liposarcoma.^[1]^ According to the 2013 edition of the World Health Organization Classification of Tumors of Soft Tissue and Bone, lipomas are categorized into 11 types.^[2]^ Spindle cell lipoma is a benign lipomatous tumor, a distinct group of lipomas composed of spindle cells, adipocytes, and multinucleated giant cells associated with ropey collagen. Immunohistochemically, spindle cell lipoma is characterized by the diffuse expression of CD34.^[3]^ Recently, CD10 expression^[4]^ and loss of nuclear expression of retinoblastoma protein have been reported.^[5]^ Spindle cell lipoma often occurs as an asymptomatic, slow-growing subcutaneous tumor in the upper back, back neck, and shoulders of elderly men,^[1]^ including unusual locations such as the leg, perineum, groin, buttocks, and foot fingers, etc.^[6]^ However, reports of spindle cell lipoma in the hypopharynx are extremely rare. The clinical and pathologic features of hypopharyngeal spindle cell lipoma based on findings from the present case combined with previously published data are summarized in Table 1.^[7–14]^ It occurs most commonly in males between the ages of 40 and 60,^[15]^ as a result of the frequent detection of androgen receptors in this tumor tissue. Here, we report a rare case of a 56-year-old man with a giant spindle cell lipoma occurring in the hypopharyngeal space, which originated in the posterior pharyngeal wall corresponding to the pyriform fossa. The literature was reviewed to elucidate the clinicopathological and pathological characteristics of this rare tumor.

**Table 1:** Features of 9 cases of spindle cell lipoma of the hypopharynx, the present case combined with previously published data.

Reported time/ref	Sex/Age	Location	Size	symptom	Follow-up	Immunohistochemistry
2001/^[7]^	Female/77Y	Pyriform sinus (Right)	3.5 × 2 cm	Dysphagia	18mo	NG
2007/^[8]^	Female/62Y	Vallecula (Left)	17 × 4 cm	Acute stridor after cough	18mo	CD34(+), bcl-2(+), CD99(+), S100(−), SMA(−)
2011/^[9]^	Female/52Y	Pyriform sinus (Left)	6 × 0.6 cm	feeling of thickness in the throat, and a mass in the mouth while coughing.	6mo	CD34(+), Vimentin(+), S100(−), CDK4(−), MDM2(−)
2012/^[10]^	Male/66Y	Circumpharyngeal area	0.6 × 0.25^∗^0.15 cm	progressive dysphagia	12mo	CD34(+), S100(−), Ki-67(+)
2013/^[11]^	Male/53Y	the lateral wall of the right pyriform sinus	5 × 3 × 0.5cm	mild dyspnea on exertion	28mo	CD34(+)
2013/^[12]^	Male/65Y	Vocal cord (Left)	NG	hoarseness, choking spells, stridor, and dyspnea	24mo	CD34(+), S100(−), actin(−), cytokeratin(−)
2015/^[14]^	Male/52Y	Larynx	7 × 5 cm	Acute stridor and dyspnea	NG	CD34(+), S100(−), Ki-67(+)
2016 (DFML)/^[13]^	Male/38Y	Pyriform sinus (left)	3.4 × 3.4 × 2.8 cm	progressive dysphagia	NG	Vimentin(+), CD34(+), Bcl-2(+), desmin(−), alpha smooth-muscle actin(−), S-100(−), ki-67(−)
Present case	Male/56Y	Posterior wall of hypopharynx corresponding to Pyriform fossa (Left)	16.86 × 2.78 × 2.38 cm	Asymptomatic	5 mo	CD34 (+), CK epithelial cells(+), SMA(+), CSM(+), Ki-67(+), S-100(−), HMB45(−), MElanA(−), CD117(−), Dog-1(−), Myogenin/MyoD1(−), Desmin(−)

DFML = dendritic fibromyxolipoma, MDM2 = mouse double minute 2 homolog, SMA = smooth muscle actin

## 2 Case presentation

**Figure 1:**
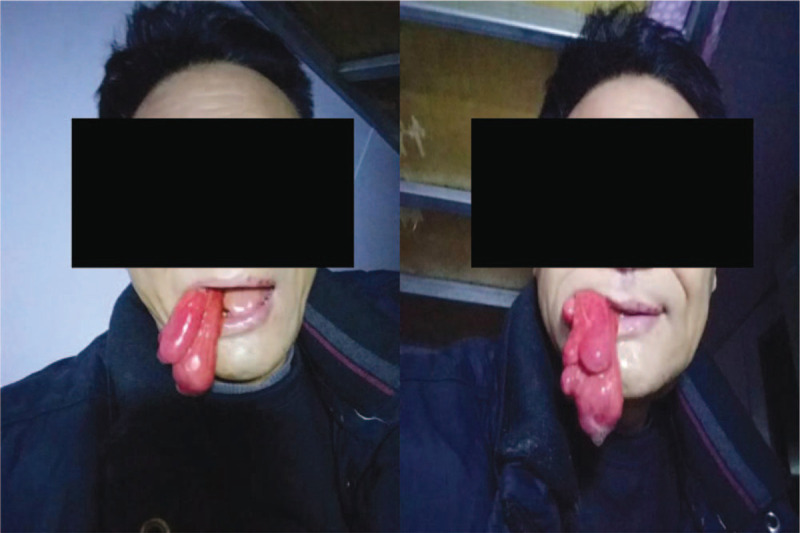
A 56-year-old male patient vomited out a smooth mass in the oral cavity provoked by intra-abdominal pressure increase after vomiting, which turned dark red over time.

**Figure 2:**
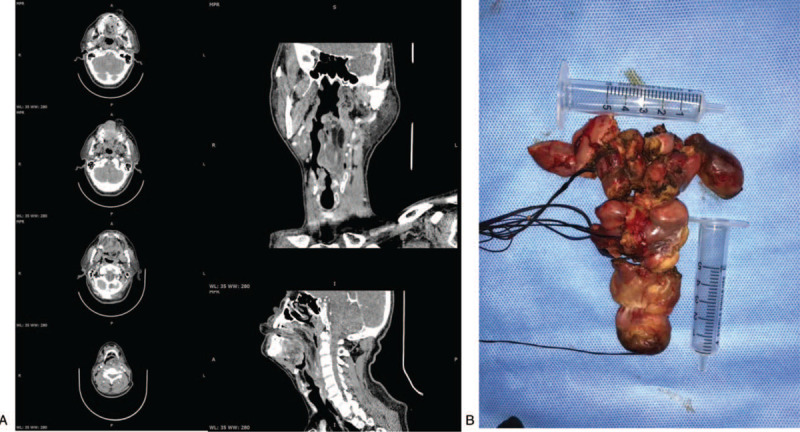
A. A high-definition CT scan showed that the tumor extending from left pyriform to oral cavity, the size of tumor was 2.38 × 2.78 × 16.86 cm. B. Because the tumor size was too large, the surgeon resected the tumor in stages with a Groff knife. The tumor was solid and rich in structurally abnormal vascular structure. CT = computed tomography.

**Figure 3:**
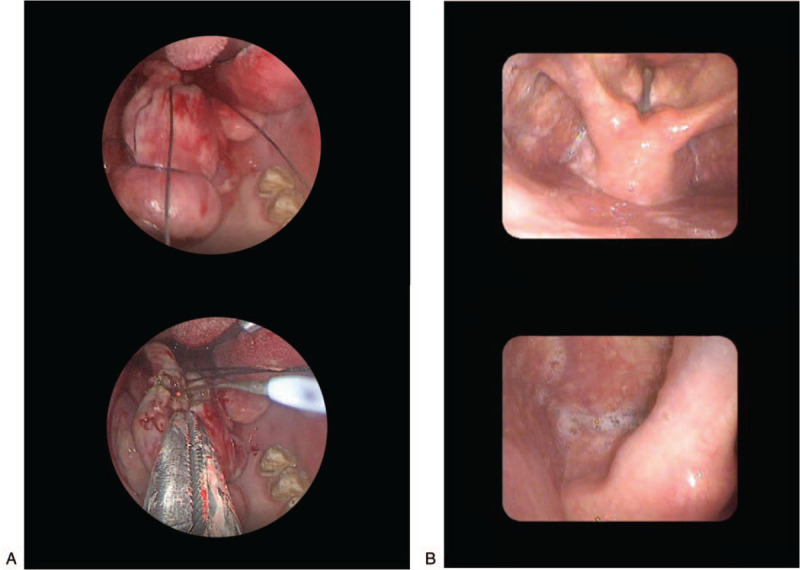
A. The patient underwent hypopharyngeal mass resection by trans-oral suspension laryngoscope. The pedicle of elongated mas was soft consistency, approximately 17 to 21 cm in length. B. Five months later, the patient visited the hospital for re-examination. The flexible fiberscope showed postoperative changes in the hypopharynx: smooth mucosa of bilateral aryepiglottic and epiglotitic fold, smooth mucosa of the bilateral pyriform fossa, and no secretion retention.

**Figure 4:**
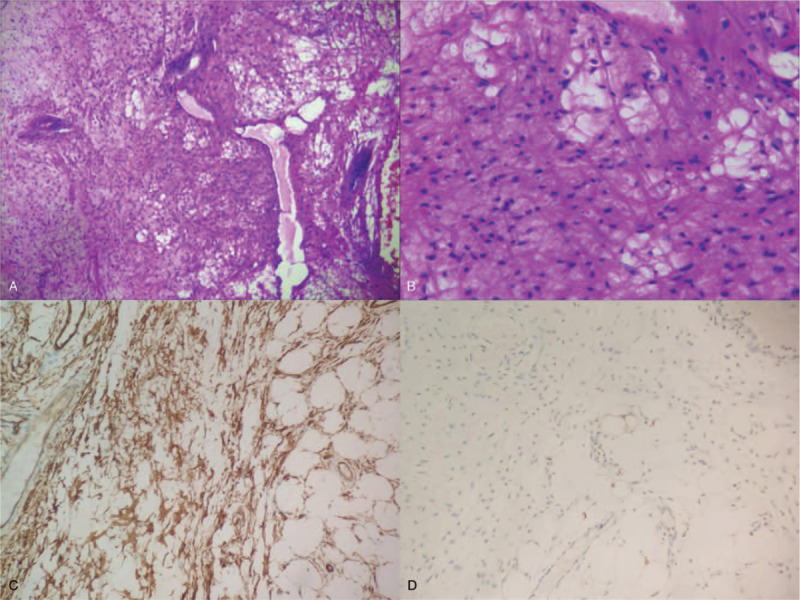
A. B. The pathological examination report showed the tumor was locally covered with squamous epithelium and focal mild atypical hyperplasia; the tumor was mainly composed of hyperplastic adipocytes, admixed with small blood vessels and a small amount of fibrous tissue. C. The immunohistochemical profile revealed positivity of spindle cells for CD34. D. The immunohistochemical profile revealed negativity for S100.

A 56-year-old male patient complained of vomiting of a smooth and giant mass in the oral cavity provoked by an intra-abdominal pressure increase after vomiting (Fig. 1). The lesion had appeared 4-years ago, growing very slowly and without any accompanying symptoms. Oral examination revealed an elongated mass protruding out of his mouth, 2.7 cm in maximal diameter. It was firm, nontender, nonfluctuant on palpation, and not attached to the surrounding or deeper tissues. The overlying mucosa was normal and turned dark red over time. A high-definition CT scan was performed which showed that the tumor extending from left pyriform to oral cavity, the size of tumor was 2.38 × 2.78 × 16.86 cm. (Fig. 2A) The flexible fiberscope was performed, showing that the pedicle of elongated mass originated from posterior wall of the hypopharynx corresponding left pyriform fossa, with soft consistency, approximately 17 to 21 cm in length. No edema was observed in the epiglottis. Routine laboratory examination showed an increase in white blood cell count (16.8 × 10^9^/L), neutrophil absolute value (13.90 × 10^9^/L), and percentage (82.7%). The results of the urine routine showed that 2 + glucose, 1 + ketone body, 1 + albumin, and urine specific gravity increased (1.039). After admission, the patient's white blood cells continuously increased, and the color of the tumor gradually turned deep red, indicating that the tumor was incarcerated, and emergency surgery was needed immediately. The patient underwent hypopharyngeal mass resection using a trans-oral suspension laryngoscope (Fig. 3A). Because the tumor size was too large, the surgeon resected the tumor in stages with a Groff knife (Fig. 2B). The tumor was solid and rich in structurally abnormal vascular structures. Histopathologically, the tumor was locally covered with squamous epithelium and focal mild atypical hyperplasia, and was mainly composed of hyperplastic adipocytes, admixed with small blood vessels and a small amount of fibrous tissue, with infiltration of acute and chronic inflammatory cells and local erosion. The surface interstitial vessels were highly ecchymosis and focal hemorrhage, inside the adipose tissue differentiated more mature, and a small number of immature short spindle cells were scattered (Fig. 4A and B). The immunohistochemical profile revealed positivity of spindle cells for CD34 (Fig. 4C), negativity for S100 (Fig. 4D), and a low proliferation with Ki-67, which confirmed the diagnosis of spindle cell lipoma and revealed its benign behavior. The epithelial cells were positive for CK, smooth muscle was positive for SMA, and calponin. The postoperative course was uneventful, and the white cell count decreased significantly to baseline on day 3 after resection. At the follow-up 5 months after operation, there was no recurrence in the left piriform fossa and no secretion retention under flexible fiberscope (Fig. 3B).

## 3 Discussion and conclusion

The incidence of lipoma is 2% in the general population, and spindle cell lipoma accounts for 1.5% of lipomas, which are rare benign lesion.^[16]^ Spindle cell lipoma often occurs in the posterior neck, upper back, and shoulders of older men.^[1]^ In addition, the long-term asymptomatic course of the disease makes diagnosis more difficult. Spindle cell lipomas originating from the hypopharynx are rare. To our knowledge, only 24 cases of spindle cell lipoma involving the hypopharynx have been reported in PubMed. We present a case of spindle cell lipoma occurring in the hypopharyngeal space, with protrusion of such with an intra-abdominal pressure increase. According to existing reports, these are described as slow-growing solitary masses, which can present with symptoms such as dysphagia, dysphonia, stridor, and foreign body sensation. However, the patient's long-standing symptoms are nonspecific; as a result, patients often feel no discomfort and take no initiative to see a doctor. Compared with a previous patient with spindle cell lipoma of the hypopharynx, the tumor size of this patient was larger; in this case, the mass was 16.86 cm in length and the white blood cells increased sharply after admission, the tumor was incarcerated and emergency surgery was needed.

Pathologically, spindle cell lipomas are composed of abundant mature adipocytes mixed with collagen-forming spindle cells. Single-vesicular or poly vesicular adipocytes and eosinophilic collagen bundles can be seen in varying numbers. Some studies have suggested the origin of spindle cells, such as fibroblasts, adipocytes, immature mesenchymal cells, and CD34-positive dendritic interstitial cells.^[16,17]^ In fact, spindle cells resemble fibroblasts with elongated nuclei, which are positive for CD34. S100 may stain mature adipocytes, but reactivity is usually not present in spindle cell lipoma.

Because there are multiple variant subtypes of spindle cell lipoma, pathologists should avoid misdiagnosis when encountering spindle cell-associated soft tissue tumors. The spindle cell lipoma cell size was significantly different from that of other benign lipomas. Focal heteromorphism of the fat nucleus and deep staining of the nucleus are helpful for diagnosis. Liposarcoma is usually a large, generally deep, painless, gradually growing mass that is more likely to occur in the lower extremities, shoulders, and retroperitoneal areas. Because of the similar clinical presentation, it is difficult to distinguish spindle cell lipoma from liposarcoma without a pathology test. The greatest morphological overlap between them was the same karyotypic alterations. The presence of adipocytes in variable amounts increases the resemblance. Pathologically, spindle cell lipoma is mainly composed of mature adipocytes mixed with uniform small spindle cells and eosinophilic collagen bundles, while liposarcoma is composed of relatively mature and hyperplastic adipose tissue. The number of single-vesicular or polyvesicular adipocytes can be seen in varying numbers. Compared with benign lipomas, the cell size of liposarcoma was significantly different. Focal heteromorphism of the fat nucleus and deep staining of the nucleus are helpful for diagnosis. In contrast to liposarcomas, spindle cell lipomas are characterized by cellular uniformity, lack of lipoblasts, and cellular pleomorphism. Immunohistochemical staining showed that the spindle cell lipoma's vimentin and CD34 were positive and S100 protein was negative, while liposarcoma's S100 protein and vimentin were often expressed, CD34 was scattered positive, and specific factors mouse double minute 2 homolog and cyclin-dependent kinase 4 were expressed.

In summary, spindle cell lipomas arising in the hypopharyngeal region are an uncommon variant of lipoma. The present case leads to further study of the clinical and pathologic characteristics of this tumor. The histology of spindle cell lipoma is distinctive, but may be easily misdiagnosed as other benign or malignant lipomatous neoplasms. Ideally, spindle cell lipomas are best managed by complete excision under endoscopy. Because it is at risk of recurrence, endoscopic follow-up of these lesions has been recommended for many years.

## Author contributions

**Conceptualization:** Zheng Liang.

**Data curation:** Hui Cao.

**Formal analysis:** Zhibin Jing, Yujie Zhang.

**Funding acquisition:** Zheng Liang.

**Writing – original draft:** Zheng Liang, Yiqing Zang.

**Writing – review & editing:** Zheng Liang, Huifang Zhou.
